# Higher and Middle Management Perspectives on Patient-Centered Care in an Oncology Setting: A Qualitative Study

**DOI:** 10.3390/nursrep14040244

**Published:** 2024-11-05

**Authors:** Majeda A. AL-Ruzzieh, Yahia M. AL-Helih, Anas Haroun, Omar Ayaad

**Affiliations:** 1 Nursing Department, King Hussein Cancer Center, P.O. Box 1269, AL-Jubeiha, Amman 11941, Jordan; ya.15238@khcc.jo (Y.M.A.-H.);; 2 Quality and Accreditation Department, Sultan Qaboos Comprehensive Cancer Care and Research Center, University Medical City, P.O. Box 566, Muscat 123, Oman

**Keywords:** patient-centered care, qualitative, higher and middle manager, oncology

## Abstract

Background: Patient center care (PCC) plays a crucial role in achieving the organizational and patient outcomes. Literature suggests that PCC enhance patient’s adherence to treatment, lower the cost of health care organization. This study aimed explore the higher and middle managers’ perceptions of patient-centered care (PCC) in an oncology setting, identify the PCC implementation challenges in the oncology setting, and understand the role of managers in facilitating PCC within the oncology context. Methods: Sampling involved the use of the purposive sampling technique on 17 middle managers and 6 upper managers who had been working in an oncology setting. The six-step thematic analysis method was used for data analysis. Results: The interviews identified six main themes and seventeen subthemes: “comprehensive care, partnership, and informed decision-making”, “infrastructure and support systems”, “leadership influence on patient-centered care”, “cultural and operational barriers”, “perceived outcomes”, and “strategies to enhance PCC in oncology”. The subthemes of comprehensive care included fostering partnerships and enabling informed decisions. The infrastructure and support systems encompassed educational empowerment and service integration. Leadership influence highlighted the role of elevating awareness, providing organizational support, and promoting comprehensive care. The cultural and operational barriers addressed the challenges faced in implementation. The perceived outcomes focused on the results of patient-centered care, while strategies to enhance PCC in oncology provided actionable insights for improvement. This provides a comprehensive understanding of the perceptions of middle and higher managers on patient-centered care (PCC) in oncology settings. It identifies key challenges in PCC implementation, highlights the critical role of managers in facilitating PCC, and offers actionable strategies for enhancing PCC.

## 1. Introduction

Patient-centered care (PCC) has become increasingly significant in modern healthcare, particularly in how hospitals approach patient treatment. It represents a paradigm shift, focusing on placing patients at the core of the healthcare system [1]. The emphasis is on addressing patients’ needs, preferences, and experiences during treatment [2]. In oncology care, this approach underscores the importance of respecting the patient’s values, fulfilling their needs, and involving them in shared decision-making [3]. By deeply understanding and prioritizing patients’ perspectives healthcare providers can deliver more compassionate and effective care, leading to improved clinical outcomes [4].

The concept of PCC was first described by Balint (1969) as “understanding the patient as a unique human being” [5], whereas it was further elaborated by the Institute of Medicine (IOM), which described PCC as “care that is respectful of and responsive to individual patient preferences, needs, and values, and that ensures that patient values guide all clinical decisions” [6]. Decades later, PCC gained more prominence as it helped increase patient satisfaction and enhance outcomes [7]. Nowadays, PCC is considered a mode for the delivery of patient care [8].

Many studies have correlated PCC as a healthcare delivery mode that improves outcomes, such as total health outcomes [9], enhanced patient adherence to medication [10], decreased healthcare utilization [11], improved healthcare quality, and enhanced rating of the care [9]. The World Health Organization has advocated PCC as a fundamental element of high-quality care [12,13] and has highlighted it as a crucial priority for healthcare improvements [14,15]. Furthermore, PCC has been incorporated into policies in developing countries [16,17].

Healthcare institutions have recently extensively adopted PCC, as evidenced by the integration of patient-centered principles into their care delivery process, as well as their vision and mission [8,18]. With the growing incorporation of PCC in healthcare settings, it is crucial to explore healthcare providers’ perceptions of PCC principles. Understanding these perspectives can illuminate potential barriers to implementation and enhance our understanding of the challenges faced by oncology healthcare workers regarding PCC [19]. Notably, the research on oncology healthcare providers’ perception of PCC remains limited, highlighting a significant gap in the literature [11,19,20,21,22,23,24].

Only two studies have assessed the perception of PCC. One of them assessed middle managers’ and front-line staff’s perceptions of PCC [11]. Qualitative interviews were conducted with front-line staff, middle managers, senior medical leadership, and other staff to assess how hospital employees conceptualized PCC. Three separate conceptualizations were identified according to the participants’ perspectives. These conceptualizations included alignment with PCC concepts surrounding the clinical encounter, extending PCC concepts into the entire patient experience and the organizational culture, and practicing traditional patient care practices [11]. The second study evaluated nurses’ understanding of PCC, employing a qualitative action research approach that classified the PCC perception into both positive and negative themes. The positives were outcome-driven healthcare, valued care providers, communication to sharpen care, and driven healthcare service, whereas the negatives were a poor approach by the nurses and the lack of an enforcement agency [24].

Currently, to the authors’ knowledge, no studies have specifically evaluated the perception of PCC among hospital employees in the oncology setting, particularly from the middle and higher management perspectives. Although managers and nurses, among others, have been asked about their views on PCC in previous studies, there is still a big need to learn more about how these views play out in oncology-specific settings [11,22,24,25].

## 2. Methods

### 2.1. Design

This study adopted a qualitative approach to collect data through one-to-one interviews to comprehensively assess the middle and higher managers’ perceptions of PCC. This approach was used to gather the data and gain an in-depth understanding of the participants’ perceptions of PCC, in addition to providing more confidentiality for such levels of management and more flexibility [26]. In addition, the participants were able to express their thoughts in a better way than they could with closed-ended questions. We used the Consolidated Criteria for Reporting Qualitative Research (COREQ) checklist for reporting this research [27].

### 2.2. Participants and Setting

We employed a purposive sampling technique to select participants, specifically targeting 17 middle managers and 6 higher managers with diverse backgrounds in oncology management. The sampling strategy aimed to achieve a mix of expertise and experience levels, as well as a variety of ages, which was essential for capturing a comprehensive view of PCC from different managerial perspectives. We contacted the participants by sending them an email invitation to participate in this study. This study was conducted in Jordan. Since a qualitative method was used, once the data were saturated, participant enrollment was ended [28].

### 2.3. Ethical Consideration

The institutional review board approved this study with approval number 20KHCC200F, following the guidelines of the Declaration of Helsinki. In response to the precautions and lockdown imposed by the COVID-19 pandemic, the IRB committee requested and subsequently approved a consent waiver for hard copies. Therefore, the authors received verbal consent from the participants, who certified that they understood the research objective and agreed to voluntarily participate. Moreover, the interviews were conducted after explaining the intent and method of this study to the higher and middle managers. The research team ensured in the instruction introduction that participation in the interviews was completely voluntary, and these participants also provided verbal consent after being informed of the research objective. Moreover, the authors informed the participants that the results would only be used for this research, that all data would be processed anonymously, and that they could refuse to answer questions or stop the interviews entirely whenever they wished.

### 2.4. Data Collection

The online interviews were conducted by a male researcher with a Ph.D. and expertise in qualitative studies. The researcher had no relationship with the participants. These interviews took place between July and October 2021 using the Zoom application, with each session lasting approximately 20–30 min. Data were collected through one-on-one semi-structured interviews, guided by questions developed from a review of the relevant literature [7,16,22,23,24]. The research questions were as follows: (1) what do you think about when you hear the term “patient-centered care?” (2) What are the key elements of patient-centered care, from your perspective? (3) What are the main facilitators to providing patient-centered care in an oncology setting, from your perspective? (4) What are the main barriers to providing patient-centered care in an oncology setting, from your perspective? (5) What are the advantages of providing patient-centered care in the oncology setting, in your opinion? (6) In your opinion, what are the benefits of providing patient-centered care to healthcare providers in an oncology setting? (7) In your opinion, what are the advantages of providing patient-centered care for organizational outcomes in an oncology setting? (8) How can we improve patient-centered care in an oncology setting?

The participants’ age, experience, profession, and working departments were obtained at the beginning of the interview. All the interviews were audio recorded on the Zoom application, version 5.0 (Zoom Video Communications, San Jose, CA, USA) and conducted by the same researcher; the entire audio recording was transcribed verbatim by a research assistant. To ensure the accuracy of the transcription, the researcher cross-checked a sample of the transcripts against the original audio recordings. Two researchers conducted the data coding. The second author performed the initial coding, and the third researcher reviewed the codes to ensure accuracy and consistency.

### 2.5. Data Analysis

For this study, the qualitative data analysis followed Braun and Clarke’s six-step thematic analysis method [29]. These steps were applied to ensure a comprehensive, thoughtful, and systematic description of the themes and subthemes within this research [29,30]. The six steps include becoming familiar with the collected data, creating initial codes, identifying themes, reviewing them, defining and refining them, and finally generating the report that guided this study.

In our methodological approach, we employed open coding in which individual lines from the transcript were treated as singular units for analysis. Words, sentences, and paragraphs that aligned with the objectives of this study were extracted. After that, similar content and dimensions were categorized as one unit, and a secondary analysis was obtained to derive the subtopics. Then, additional categories were added based on the new interview data. Data saturation was achieved within 23 interviews.

We followed Guba and Lincoln’s four issues: credibility, applicability, consistency, and neutrality [31] to ensure the rigor of this study. To ensure credibility, detailed information from participants was collected without bias or preconceived ideas. The applicability of the findings was assessed by checking their relevance and practicality in various scenarios, aligning with the managers’ experiences. This study employed theoretical sampling, including diverse participant backgrounds, to prevent bias. Consistency was guaranteed by coding key concepts and ensuring the replicability of this study. The coding involved marking important words and phrases, abstracting categories, and analyzing raw data for reliability. The researchers continuously discussed and validated the findings, improving reliability and validity. Lastly, neutrality was maintained by ensuring the absence of bias when credibility, applicability, consistency, and neutrality were established. Overall, this study aimed for rigor by meeting these criteria.

## 3. Result

This study involved 17 middle managers and 6 upper managers. There were 11 male and 6 female middle managers, with a mean age of 40 years (ranging from 29 to 53). Among them, 2 were directors and 15 were managers, with an average of 15 years of experience. Additionally, six higher managers participated, with an average age of 50 years (ranging from 44 to 59), including five males and one female. All the higher managers held chief positions, with an average of 23 years of experience.

### 3.1. Themes

Table 1 illustrates the main themes and subthemes of this study. Six main themes and seventeen subthemes were identified: comprehensive care and partnership; facilitating a patient-centered internal organizational environment; cultural, attitudinal, and operational barriers; perceived outcomes; and strategies to enhance patient-centered care (PCC) in an oncology setting (Supplementary Materials Table S1).

#### 3.1.1. Theme 1: Comprehensive Care, Partnership, and Informed Decision-Making

All the interviewees emphasized the significance of patient-centered care (PCC), asserting that the primary consideration when discussing PCC was respecting patients’ preferences and needs. “*PCC is care that depends on a partnership between patients and their families from one side and health care providers from the other side to provide optimal care based on patients’ needs, preference, and values*” *(P03)*. Moreover, the participants emphasized the significance of patient and family involvement in the treatment journey. Meanwhile, some of this study’s participants mentioned partnership and sharing decisions between the physicians and the patients, “*when all the center departments are working together and integrally to achieve the optimal care of patients, the patient is the core of any service*” (P13). Additionally, the participants stressed the importance of personalized medicine, customized care, and tailored healthcare services. The first word that came to mind when considering PCC for six middle managers and two higher managers was comprehensive and integrated care “*Comprehensive care for all aspects, providing treatment, taking care of his social life, and the involvement of his family*” *(P11).* Meanwhile, five middle managers emphasized the importance of partnership and effective communication between healthcare professionals and patients. Three subthemes emerged from the comprehensive care and partnership theme*:* (i) respecting patient preferences and needs, (ii) patient and family involvement throughout treatment, and (iii) partnership and shared decision-making.

#### 3.1.2. Theme 2: Infrastructure and Support Systems

The results indicate that the middle managers and higher managers considered the acceptance of the PCC concept by healthcare providers (HCPs) as a crucial element. The middle managers noted that the patient’s level of education plays an important role in PCC, “*the key element is the level of education of the patient and his family; if they are well educated, that will make PCC much better*” *(P02)*. Physical and psychological comfort were emphasized by the managers for effective PCC implementation. Information availability and accessibility were described as critical key elements. The participants viewed the integration of services and resources within the organization as a crucial element. “From my perspective, I think that the key elements of PCC could be: “* patient and family involvement in decision-making; *availability of information to the patient and updating him with any case progression; *all the resources of the organization should be directed to achieve best-provided service with minimum patient suffering” *(P19).* Two subthemes emerged from the infrastructure and support systems theme: (i) educational empowerment and information accessibility, and (ii) comfort and integration of services.

#### 3.1.3. Theme 3: Leadership Influence on Patient-Centered Care

The participants of this study indicated that spreading awareness of PCC among patients and medical staff is one of the main facilitators of PCC in oncology settings. “*It needs dedication; start doing real projects for PCC; open discussion between patients and HCP since patient and HCP perception is very important. It takes knowledge and time to implement and maintain PCC. Regarding patient awareness about PCC, it is a very important issue*” *(P05).* Moreover, the participants discussed how leadership plays a significant role in raising awareness of PCC at various levels through awareness programs and PCC enculturation. “*Oncology leadership, from here we begin, how they feel toward implementing PCC; presence strong vision, support, follow up, and establishing of PCC awareness program and PCC enculturation at level of health care providers, patients, and community, this will lead to be implemented easily*” (P01).

The majority of the managers addressed supportive administration, strong vision, and mission as key elements of influencing PCC. An open discussion with patients could be one of the facilitators for effective implementation: “*HCPs’ and patients’ awareness via open discussion between patients themselves and patients with HCPs. Also, you could spread awareness via TV advertisements*” (P06). While some middle managers believed that financial support and good infrastructure were important, the participants also reported the presence of a variety of treatment options, multidisciplinary conferences (MDCs), and effective communication between HCPs and patients as facilitators. “*The treatment guidelines in our center are going to facilitate the implementation of PCC. In addition to the variety of available treatments in our center, so many options could be discussed with the patient, and MDC (multidisciplinary care) plays an important role in PCC, which helps to explain the most appropriate treatment to the patient in a perfectly scientific way*” (P12).

Three subthemes emerged from the leadership influence on patient-centered care theme: (i) elevating awareness and effective communication strategies; (ii) organizational support, leadership, and resources; and (iii) promoting comprehensive care and collaborative practices.

#### 3.1.4. Theme 4: Cultural and Operational Barriers

The middle and higher managers participating in this study reported culture as one of the main barriers to the patient having ultimate physician trust, so some patients do not prefer to be involved in deciding the treatment plan and do not easily accept the concept of PCC. “*One of the barriers to PCC was our culture. Some patients still have a paternalistic aspect of care, which means they believe that the physician is highly trained and educated with great experience, and so his decision is the best choice regardless of what the patient himself prefers. In our patients’ perceptions, physicians are highly trusted*” *(P02)*. Moreover, the middle managers highlighted that the education level of the patients is also considered as a barrier to delivering PCC. While communication difficulties could prevent the proper implementation of PCC, eight middle managers and two higher managers identified work overload, lack of time, and the clinic workflow as barriers. “*There is high turnover, so the new employee cannot be impeded by the new culture of PCC easily*” *(P17).* “*Also, time could be a barrier, like the workflow of the clinic, when we need more time to be spent with the patient, and further education is needed. PCC is new, so there’s still a lack of clarity about what the patients and staff need. Communication difficulties might be challenging*” *(P01).* Furthermore, the middle managers agreed that high staff turnover was an obstruction, in addition to the nature of the disease. Meanwhile, the majority of the participants highlighted that finance and space obstacles could be barriers to PCC. In some cases, restrictive treatment guidelines prompted the HCP to implement PCC. The theme of cultural and operational barriers gave rise to three subthemes: (i) trust and confidence, (ii) patient education and awareness, and (iii) work overload, time constraints, and clinic workflow.

#### 3.1.5. Theme 5: Perceived Outcomes

The interviewees totally agreed on the positive influence of PCC on patients’ outcomes. The majority of the participants noted that PCC significantly boosted patient satisfaction by enhancing their treatment experiences. Additionally, they observed that PCC played a vital role in increasing patient compliance with treatment plans, ultimately resulting in higher survival rates. Moreover, the participants identified an overall improvement in treatment outcomes due to the implementation of PCC. Definite, better curing and a better treatment outcome, in addition to patient satisfaction, “*would ensure compliance with the treatment*” (P09). Furthermore, the beneficial effects of PCC on enhancing patients’ psychological comfort, safety, and communication with healthcare providers was highlighted, ultimately contributing to shorter hospital stays.

Furthermore, the results strongly suggested that PCC also significantly impacted the healthcare providers’ outcomes. All the interviewees acknowledged the positive effect of PCC on healthcare providers. The participants agreed that PCC streamlined the process of choosing treatment plans, making it easier for healthcare providers to navigate “*definitely, better curing, and better treatment outcome, in addition to patient satisfaction, so he would comply with the treatment*”*(P09).* Additionally, they emphasized increased satisfaction among healthcare providers when PCC principles were implemented “*Satisfied HCPs, more qualified HCPs will be obtained with PCC*” *(P10)*. Furthermore, the middle managers highlighted that PCC could positively influence the reputation of healthcare facilities. “*Achieving a reputation for the HCP when the patient feels that he is in the center of care and he decides his type of treatment, especially an educated patient*” *(P01).* Moreover, the managers noted that PCC enhances professionalism among healthcare providers, fostering confidence and motivation. It was reported by some of the middle managers that this heightened professionalism even led to increased staff retention, demonstrating the far-reaching impact of PCC on the healthcare providers’ well-being and on the organizational environment. “*From my point of view, PCC could lead to a higher level of staff satisfaction, in addition to the easier decision-making process, due to the lower patient resistance regarding the treatment plan*” *(P14).*

Regarding the organizational benefits derived from PCC, all the interviewees addressed its positive impact. Almost all the managers participating in this study underscored that the cancer center’s excellent reputation could draw patients not only locally but also internationally. Some of them even addressed that it could attract experts to the center. Financially, PCC affected the income positively, as specified by three middle managers. “*PCC will improve the outcomes because of the higher recovery rate and curing, so the center gains a great reputation*” *(P19).* Three subthemes emerged from the perceived outcomes theme: (i) patient outcomes, (ii) healthcare provider outcomes, and (iii) organizational outcomes.

#### 3.1.6. Theme 6: Strategies to Enhance PCC in an Oncology Setting

The participants in this study stated that the best way to improve PCC was by spreading awareness among the patients and medical staff, while some of them agreed that willingness and support from the administrators was also important. The middle managers suggested that focused group discussion with patients is one of the ideas to boost PCC. “*Doing more intense focused group discussion, and hearing from patients, should be in all KHCC departments, and try to cover patients’ diversity*” *(P05)*. The participants offered an alternative perspective, proposing a tool as a quality indicator to facilitate feedback analysis: “*Prepare a proper indicator to figure out the perfect implementation of PCC and the presence or absence of the key elements. And compare that with other organizations where PCC is implemented*” *(P10)*. Some managers found that medical research, training programs, and listening to patient voices are credible ways to promote PCC: “*Patient and family voice are the best way to improve PCC, where you can know the gaps*” *(P19)*. Three subthemes *emerged from the strategies to enhance PCC in oncology theme: (i) structural strategy, (ii) process strategy,* and *(iii) outcome strategy.*

## 4. Discussion

The findings revealed six important themes, including comprehensive care and partnership; infrastructure and support systems; facilitating a patient-centered ecosystem; cultural, attitudinal, and operational hindrances; perceived outcomes; and strategies to enhance patient-centered care (PCC) in an oncology setting, with seventeen subthemes.

### 4.1. Theme 1: Comprehensive Care, Partnership, and Informed Decision-Making

This study’s findings emphasize the crucial role of PCC in enhancing healthcare delivery through comprehensive care, partnership, and informed decision-making. The managers’ emphasis on respecting patient preferences and needs reflects a growing recognition within healthcare management of the necessity to prioritize the patient’s voice, in line with the existing literature [32,33,34,35]. This focus is crucial in fostering a collaborative environment that values the preferences of patients and their families, leading to more effective care, which is in line with the recent literature [32,33,34]. This emphasizes a paradigm shift toward a collaborative-care model.

Furthermore, this study demonstrates the perception of personalized care as a fundamental element of PCC. The emphasis on tailored healthcare services ensures that care is not only comprehensive but also relevant to the patients’ circumstances, acknowledging the diverse needs of patients and advocating that the healthcare system is responsive rather than one size fits all. Personalizing care can significantly impact the efficacy of the provided care and the holistic well-being of the patient. This aligns with the existing literature [36,37]. In practice, this study suggests several actionable strategies for implementing PCC. Training programs that emphasize the significance of PCC, along with tools that facilitate shared decision-making, and the development of policies that actively promote family involvement in decision-making could contribute to effective PCC, particularly in oncology settings, and strengthen the partnership model.

### 4.2. Theme 2: Infrastructure and Support Systems

The study participants’ insights reveal several critical components essential for effective PCC implementation in oncology settings. HCPs’ acceptance of the PCC concept is considered one of the critical components for effective implementation; similarly, the level of the patient and his family’s education is crucial for effective PCC implementation, aligning with the prior literature [1,38,39]. This underlines the need for educational initiatives aimed at empowering patients to engage actively in their care process. While physical and psychological comfort are crucial elements, a comfortable care environment significantly influences patient satisfaction and engagement [40]. Information accessibility and availability were also identified as key elements [41]. Thus, the integration of resources and services emphasizes the need for a cohesive approach to provide comprehensive care and reduce patient suffering through patient and family involvement in decision-making. This is in line with the prior literature conclusions [24,42,43].

### 4.3. Theme 3: Leadership Influence on Patient-Centered Care

This study’s findings revealed that leadership plays a crucial role in developing an encouraging environment for effective PCC implementation in oncology settings. Spreading awareness and enculturating PCC at the level of patients, families, and HCPs is one of the core principles of PCC, as it fosters a culture of engagement and collaboration, which aligns with the findings of prior studies [44]. Furthermore, PCC emphasizes strong organizational vision and mission as crucial elements. It serves as a guiding framework for all healthcare providers, leading to enhanced patient care, which can foster an organizational culture that values collaboration and compassion. Leaders should actively reflect this vision in their daily operations and patient interactions [18,42,45].

Open communication between healthcare professionals (HCPs) and patients also helps create a space in which worries can be shared and treatment options can be talked over in detail, which ultimately leads to successful PCC implementation [46,47]. Meanwhile, financial support and infrastructure are considered facilitators of PCC implementation, consistent with findings in the prior literature. [44].

### 4.4. Theme 4: Cultural and Operational Barriers

This study’s findings emphasize how cultural belief can impair effective PCC implementation, as trust in physicians significantly influences patient decision-making due to the physicians’ skills and experience. The effectiveness of care significantly correlates with perceived trust in physicians [48]. Hence, patients rely totally on the physician’s decisions. Researchers identified these cultural beliefs as significant barriers to effective PCC, influencing the decision-making process in oncology [49]. Understanding cultural diversity is essential for implementing PCC, as traditional approaches to care may conflict with the collaborative spirit of effective PCC, aligning with findings from the existing literature [50,51].

Prior studies [44,52,53,54] have highlighted work overload and high employee turnover as barriers to effective PCC in oncology settings. Moreover, this study identified financial constraints and space limitations as barriers to PCC, highlighting how limited resources can hinder organizations’ ability to implement comprehensive initiatives. This aligns with the findings of a study that identified financial constraints as a significant barrier to PCC at the organizational level [44].

### 4.5. Theme 5: Perceived Outcomes

The findings strongly support the significant impact of PCC on various outcomes, including those related to organizations, HCPs, and patients. This study emphasized that PCC improved patient satisfaction by enhancing the patient experience. The results align with this, demonstrating that PCC approaches lead to higher patient satisfaction [14,55,56,57,58]. Additionally, PCC has a positive impact on organizational outcomes, including enhanced patient quality of life and hospital reputation. Studies have also revealed that hospitals practicing PCC often experience better clinical outcomes, leading to an enhancement of the hospital’s reputation [44,58,59]. Additionally, the participants emphasized that PCC is forming the process of choosing treatment plans for cancer patients; this is in line with the prior literature [60,61,62].

### 4.6. Theme 6: Strategies to Enhance PCC in an Oncology Setting

This study’s result offers valuable insights into practical steps to enhance the implementation of PCC. The agreement among the participants reveals that awareness, administrative support, patient’s involvement, quality indicators, and continuous feedback are essential components in advancing PCC in oncology settings.

The key strategy to effectively implement PCC in an oncology setting is to enhance the awareness of PCC. This strategy aids in aligning clinical practice with PCC principles, ensuring that care is not only medically appropriate but also tailored to the patient’s preferences and values. Furthermore, raising patient awareness empowers them to make informed decisions about their health. This is a key pillar of PCC [63,64]. Leaders play a crucial role in setting the vision for PCC, allocating resources, and ensuring that HCPs have the necessary tools and training to implement PCC effectively. This finding aligns with a study that found the impact of leadership in managing changes [65].

Furthermore, involving patients in group discussions and providing feedback aids in identifying gaps in care delivery, understanding diverse patient perspectives, and enhancing trust between HCPs and patients. This strategy fosters open communication, which positively impacts patient outcomes [24,32,66]. Finally, the managers addressed listening to patient and family voices as one strategy to enhance PCC in an oncology setting. This outcome strategy aids in evaluating and recognizing patient concerns, achieving the active participation of patients in managing their disease throughout the cancer journey, and enhancing the concept of continuity of care and empowering patients. All these strategies are considered the core of effective PCC in oncology settings, which is consistent with the findings of [24,44,66,67].

## 5. Conclusions

This study underscores the multifaceted aspects of patient-centered care (PCC) in oncology settings, underscoring the crucial role of comprehensive care; infrastructure support; leadership; and the need to overcome cultural beliefs, resource constraints, and staff overload to ensure effective PCC implementation. Moving forward, strategies like increasing awareness, ensuring patient involvement, and continuously refining quality indicators are necessary to strengthen PCC practices. Future research should focus on refining these strategies and exploring their long-term impact on patient outcomes and healthcare delivery in oncology.

## 6. Recommendations

Based on the findings from this study, several recommendations can be made to enhance the implementation of PCC in oncology settings:

1. Increase awareness and education: This will be through an educational program that empowers patients to actively participate in their care, fostering better decision-making and enhancing treatment. HCPs need to receive comprehensive training to effectively integrate patient preferences and values into their clinical practice.
2. Invest in strengthening leadership and administrative support, as leaders are crucial in fostering an environment that promotes PCC. Healthcare organizations should encourage leaders to prioritize PCC by allocating resources, setting clear vision, and facilitating ongoing training. Strong leadership is essential for promoting a culture of collaboration and patient engagement.
3. Enhance patient involvement: Encouraging patient participation in focused group discussions and the decision-making process can help identify gaps in care and improve the overall patient experience. Active listening to patient and family voices allows HCPs to better understand diverse perspectives and tailor care accordingly.
4. Deal with operational and cultural barriers: For PCC to work well, organizations should be aware of cultural issues and work to get past problems like doctors making all the decisions, too much work, and not enough resources.

## Figures and Tables

**Table 1:** Main themes and subthemes.

Theme	Subtheme
Comprehensive care, partnership, and informed decision-making	Respecting patient preferences and needs. Patient and family involvement throughout treatment. Partnership and shared decision-making.
2. Infrastructure and support systems	Educational empowerment and information accessibility. Comfort and integration of services.
3. Leadership influence on patient-centered care	Elevating awareness and effective communication strategies. Organizational support, leadership, and resources. Promoting comprehensive care and collaborative practices.
4. Cultural and operational barriers	Trust and confidence. Patient education and awareness. Work overload, time constraints, and clinic workflow.
5. Perceived outcomes	Patient outcomes. Healthcare provider outcomes. Organization outcomes.
6. Strategies to enhance patient-centered care (PCC) in oncology	Structural strategy. Process strategy. Outcome strategy.

## Data Availability

The datasets used and analyzed during the current study are available from the corresponding author upon reasonable request.

## Supplementary Materials

The following supporting information can be downloaded at: https://www.mdpi.com/article/10.3390/nursrep14040244/s1, Table S1: Main themes and subthemes.

## Public Involvement Statement

There was no public involvement in any aspect of this research.

## Guidelines and Standards Statement

This manuscript was drafted against the Consolidated Criteria for Reporting Qualitative Research (COREQ) checklist for reporting qualitative research [27].
