# Supplementary material for: Higher and Middle Management Perspectives on Patient-Centered Care in an Oncology Setting: A Qualitative Study
Source: Nurs Rep. 2024 Nov 5;14(4):3378–90. doi: 10.3390/nursrep14040244 (PMC11587424; doi:10.3390/nursrep14040244)
Supplement: Supplementary file 1 [file nursrep-14-00244-s001.zip › nursrep-3126104-supplementary.pdf]

Table S1. Main themes and subthemes.

	Theme/subtheme	Definition
1	**Theme 1: Comprehensive Care, Partnership, and Informed Decision-Making**	This theme emphasizes a holistic approach to patient care that is centered on collaboration between healthcare providers, patients, and their families. It highlights the importance of considering the patient's preferences, ensuring they are involved in every step of the treatment process, and promoting shared decision-making to achieve optimal outcomes.
1.1	Respecting Patient Preferences and Needs	This sub-theme focuses on tailoring care to each patient's unique needs, values, and preferences. It involves understanding and respecting what matters most to the patient, including their cultural, emotional, and physical needs, and ensuring that these preferences are integrated into the care plan.
1.2	Patient and Family Involvement throughout Treatment:	This sub-theme stresses the active participation of both patients and their families in the treatment process. It involves keeping them informed, inviting their input in decision-making, and fostering a sense of partnership in managing the patient's health. Family members, as part of the care team, provide support and contribute to decisions affecting the patient's well-being.
1.3	Partnership and Shared Decision-making:	This sub-theme highlights the collaborative relationship between healthcare providers, patients, and families in making informed decisions about treatment options. It emphasizes the importance of clear communication, mutual respect, and ensuring that patients are empowered to make choices about their care in partnership with their healthcare team.
2	**Theme 2: Infrastructure and Support Systems**	This theme centers on the importance of having strong, well-organized infrastructure and support systems that facilitate effective healthcare delivery. It includes both physical and informational systems that empower patients and healthcare providers, ensuring that care is seamless, accessible, and supportive throughout the treatment journey.
2.1	Educational Empowerment and Information Accessibility:	This sub-theme highlights the need for accessible educational resources and information for patients, families, and healthcare professionals. It emphasizes the importance of empowering patients with the knowledge needed to make informed decisions about their care, as well as ensuring healthcare providers have the resources necessary to educate and guide their patients. Information must be clear, culturally appropriate, and available in various formats to suit different needs.
2.2	Comfort and Integration of Services:	This sub-theme focuses on the physical and emotional comfort provided to patients through the integration of services across different care settings. It stresses the need for a well-coordinated approach where various services, such as medical care, psychological support, and rehabilitation, are seamlessly integrated to ensure that patients receive holistic and comfortable care. This also includes creating a supportive environment that reduces stress and promotes healing.
3	**Theme 3: Leadership Influence on Patient-Centered Care**	This theme explores the role of leadership in fostering a healthcare environment that prioritizes patient-centered care. It focuses on how leaders can shape the culture of care by

		promoting awareness, effective communication, organizational support, and collaboration, ensuring that the needs of patients are consistently at the forefront of care delivery.
3.1	Elevating Awareness and Effective Communication Strategies	This sub-theme emphasizes the leader's role in raising awareness about the importance of patient-centered care and implementing communication strategies that facilitate clear, open, and respectful dialogue between healthcare providers, patients, and families. Leaders are responsible for fostering a culture where communication is prioritized, ensuring that both patients and staff feel heard and informed throughout the care process.
3.2	Organizational Support, Leadership, and Resources:	This sub-theme focuses on the influence of leadership in providing the necessary support and resources to implement patient-centered care effectively. It highlights the importance of organizational structures that promote leadership involvement, provide adequate staffing, technology, and training, and allocate resources to ensure that care is delivered in a way that aligns with patient needs and expectations.
3.3	Promoting Comprehensive Care and Collaborative Practices:	This sub-theme underscores the role of leadership in encouraging a comprehensive, multidisciplinary approach to patient care. Leaders play a key role in fostering collaboration among various healthcare professionals to ensure that care is well-coordinated, holistic, and centered on the patient's overall well-being. They are instrumental in promoting practices that integrate different aspects of care to achieve better health outcomes and patient satisfaction.
4	**Theme 4: Cultural and Operational Barriers**	This theme focuses on the challenges that healthcare systems and professionals face in delivering effective care, especially when addressing patient needs within diverse cultural contexts and operational constraints. These barriers can impede the quality of care, affect patient outcomes, and limit the efficiency of healthcare services.
4.1	Trust and Confidence:	This sub-theme addresses the issues related to building and maintaining trust between healthcare providers and patients, especially in culturally diverse settings. A lack of trust can arise due to cultural misunderstandings, past negative experiences, or poor communication, which can lead to decreased patient confidence in their healthcare team and reduced engagement in their care.
4.2	Patient Education and Awareness:	This sub-theme highlights the barriers related to educating patients about their health conditions, treatment options, and healthcare processes. Cultural differences, language barriers, and varying levels of health literacy can hinder patients' understanding of their care, making it difficult for them to make informed decisions or fully participate in their treatment.
4.3	Work Overload, Time Constraints, and Clinic Workflow:	This sub-theme focuses on the operational challenges healthcare professionals face, such as high patient volumes, limited time with each patient, and inefficient clinic workflows. These issues can lead to rushed consultations, decreased attention to patient needs, and burnout among healthcare workers, ultimately affecting the quality of care and patient satisfaction.
5	**Theme 5: Perceived Outcomes**	This theme focuses on the various results or consequences that stakeholders in healthcare perceive as a result of interventions, processes, or care strategies. It covers the impact on patients, healthcare

		providers, and the organization as a whole, highlighting the multifaceted nature of outcomes in healthcare settings.
5.1	Patient Outcomes:	This Sub-theme addresses the effects of healthcare interventions on patient health and well-being. These outcomes include improvements in physical and mental health, quality of life, satisfaction with care, and adherence to treatment plans. It also considers negative outcomes such as complications or dissatisfaction, reflecting how care impacts patients both directly and indirectly.
5.2	Healthcare Provider Outcomes:	This sub-theme focuses on the effects that care processes and workplace conditions have on healthcare providers. It includes provider satisfaction, burnout, stress levels, and professional growth. Positive outcomes might involve job satisfaction, empowerment, and the ability to provide high-quality care, while negative outcomes can include stress, fatigue, and reduced performance.
5.3	Organization Outcomes:	This sub-theme reflects the impact of healthcare practices and strategies on the organization as a whole. It considers metrics such as operational efficiency, patient satisfaction scores, financial performance, and the institution's reputation. Organizational outcomes can be positive, such as improved productivity and reduced costs, or negative, like inefficiencies, financial losses, and declining patient trust.
6	**Theme 6: Strategies to Enhance Patient-Centered Care (PCC) in Oncology**	This theme focuses on the various approaches and strategies aimed at improving patient-centered care (PCC) within oncology settings. It emphasizes creating structures, processes, and outcomes that place patients' needs, values, and preferences at the core of care delivery, improving the quality of care and patient satisfaction in cancer treatment.
6.1	Structural Strategy:	This sub-theme refers to the organizational and physical structures that support patient-centered care in oncology. It includes building or modifying the healthcare infrastructure to ensure that facilities, resources, and technologies are aligned with PCC principles. Examples include creating dedicated patient navigation systems, ensuring proper staffing, and offering comfortable and accessible environments for cancer patients and their families.
6.2	Process Strategy:	This sub-theme focuses on the workflow and procedures that enhance PCC in oncology care. It involves implementing processes that facilitate effective communication, shared decision-making, and coordination between healthcare providers, patients, and their families. Process strategies include multidisciplinary team meetings, patient education programs, and individualized care plans that ensure the patient's voice is central to all decisions.
6.3	Outcome Strategy:	This sub-theme highlights strategies aimed at improving measurable outcomes in patient-centered care. It focuses on setting specific goals related to patient satisfaction, health outcomes, and quality of life. These strategies involve tracking and evaluating the success of PCC initiatives through feedback, patient-reported outcomes, and quality indicators, ensuring continuous improvement in the delivery of oncology care.
